# Endoscopic Management of Lemmel Syndrome Complicated by Duodenal Diverticulitis: A Case Report

**DOI:** 10.7759/cureus.100417

**Published:** 2025-12-30

**Authors:** Jorge A Garcia Garza, Julio Garza Vega, Claudia M Cardosa Gonzalez, Raymundo Zavala Salazar, Bianca S Garcia Beattie, Sergio E Nava Ramos, Angela L Juárez-Villarreal, Nicolas S Alfaro Espinoza

**Affiliations:** 1 General Surgery, Hospital Regional de Monterrey Instituto de Seguridad y Servicios Sociales de los Trabajadores del Estado (ISSSTE), Monterrey, MEX; 2 General Medicine, Universidad de Monterrey, Monterrey, MEX; 3 General Surgery, Hospital General de Zona No. 17 Instituto Mexicano del Seguro Social (IMSS), Monterrey, MEX

**Keywords:** acute cholangitis, endoscopic management, endoscopic retrograde cholangiopancreatography (ercp), lemmel's syndrome, obstructive jaundice, periampullary duodenal diverticulum

## Abstract

Lemmel syndrome is a rare cause of biliary obstruction resulting from the extrinsic compression of the distal common bile duct by a periampullary duodenal diverticulum in the absence of choledocholithiasis or malignancy. We present the case of a 62-year-old male patient with a history of hepatic steatosis and a recent laparoscopic cholecystectomy who was admitted with right upper quadrant pain and jaundice. Laboratory findings showed leukocytosis, cholestatic liver enzyme elevation, and hyperbilirubinemia consistent with severe cholangitis. Imaging revealed biliary ductal dilation without calculi or mass, and endoscopic retrograde cholangiopancreatography identified chronic inflammatory changes in the ampulla of Vater adjacent to a duodenal diverticulum. After performing a sphincterotomy and placing a biliary stent, the patient exhibited complete clinical and laboratory resolution. Histopathology confirmed chronic inflammatory changes of the periampullary mucosa. Endoscopic retrograde cholangiopancreatography continues to be the gold standard for both diagnosis and treatment. Individualized management is essential, and endoscopic therapy offers excellent results in properly selected patients.

## Introduction

First described by Gerhard Lemmel in 1934, Lemmel syndrome is defined as biliary obstruction not secondary to choledocholithiasis or malignancy but caused by a periampullary duodenal diverticulum (PDD) that exerts extrinsic compression on the distal common bile duct (CBD) [1]. Although most PDDs are asymptomatic, approximately 5% of cases may result in clinical symptoms or complications [2]. Duodenal diverticula are reported in up to 22% of individuals, and approximately 10% are situated close enough to the ampulla of Vater (AV) to cause clinically significant biliary compression [3]. PDDs may lead to obstruction through direct mechanical compression, sphincter of Oddi dysfunction, or chronic inflammation of the ampulla. Misdiagnosis is frequent, as periampullary diverticula are often overlooked on non-invasive imaging and can mimic choledocholithiasis or pancreatic neoplasms, leading patients to undergo unnecessary interventions such as cholecystectomy before the true etiology is recognized [3,4]. We report the case of a 62-year-old male patient presenting with biliary obstruction secondary to a PDD associated with acute diverticulitis involving the ampullary region, an uncommon and underreported mechanism, shortly after cholecystectomy and in the absence of choledocholithiasis or pancreatic neoplasia, successfully managed with endoscopic retrograde cholangiopancreatography (ERCP).

## Case presentation

A 62-year-old man with a history of grade III hepatic steatosis and cholecystitis, who had undergone laparoscopic cholecystectomy one week prior at an outside institution, presented to the emergency department with persistent right upper quadrant pain rated 7/10 on a numeric rating scale following a greasy meal, accompanied by marked jaundice. At presentation, the patient was hemodynamically stable and afebrile. Physical examination revealed scleral and cutaneous jaundice, tenderness on deep palpation of the right upper quadrant, and a positive Murphy's sign; the rest of the physical examination showed no abnormalities. Laboratory evaluation demonstrated leukocytosis with neutrophil predominance and clear evidence of hepatic dysfunction, reflected by a markedly elevated international normalized ratio (INR) of 13.67, direct hyperbilirubinemia, and significantly elevated transaminases (Table 1). According to the Tokyo Guidelines 2018, coagulation abnormalities, specifically an INR ≥1.5 in the absence of anticoagulation, constitute organ dysfunction and fulfill the criteria for Grade III (severe) acute cholangitis [5]. Based on these findings, the episode was classified as Grade III cholangitis, and management was initiated with broad-spectrum antibiotics and fresh frozen plasma (FFP) to correct the coagulopathy. Endoscopic intervention was deferred until clinical stabilization was achieved.

**Table 1:** Admission laboratory results The patient presented with leukocytosis, cholestatic liver enzyme elevation, and marked coagulopathy, fulfilling criteria for Grade III acute cholangitis due to organ dysfunction according to the Tokyo Guidelines 2018. Renal parameters and metabolic markers remained within normal limits. Lactate and lipase values were not available. WBC: white blood cells; TB: total bilirubin; IB: indirect bilirubin; DB: direct bilirubin; ALT: alanine aminotransferase; AST: aspartate aminotransferase; ALP: alkaline phosphatase; INR: international normalized ratio; PT: prothrombin time; PTT: partial thromboplastin time

Parameter	Result	Reference range
WBC	14,940/mm³	4,000-10,000/mm³
Hemoglobin	15.5 g/dL	14-18 g/dL
Platelets	466,000/mm³	150,000-400,000/mm³
TB	9.8 mg/dL	0.1-1.2 mg/dL
IB	1.5 mg/dL	0-1.0 mg/dL
DB	8.3 mg/dL	0-0.5 mg/dL
ALT	131 U/L	<45 U/L
AST	149 U/L	<40 U/L
ALP	793 U/L	44-147 U/L
INR	13.67	0.8-1.2
PT	151.7 sec	11.3-14.5 sec
PTT	71.2 sec	27.2-32.2 sec
Glucose	133 mg/dL	74-106 mg/dL
Urea	78 mg/dL	15-43 mg/dL
Creatinine	1.3 mg/dL	0.5-1.3 mg/dL
Amylase	38 U/L	30-110 U/L

**Figure 1:**
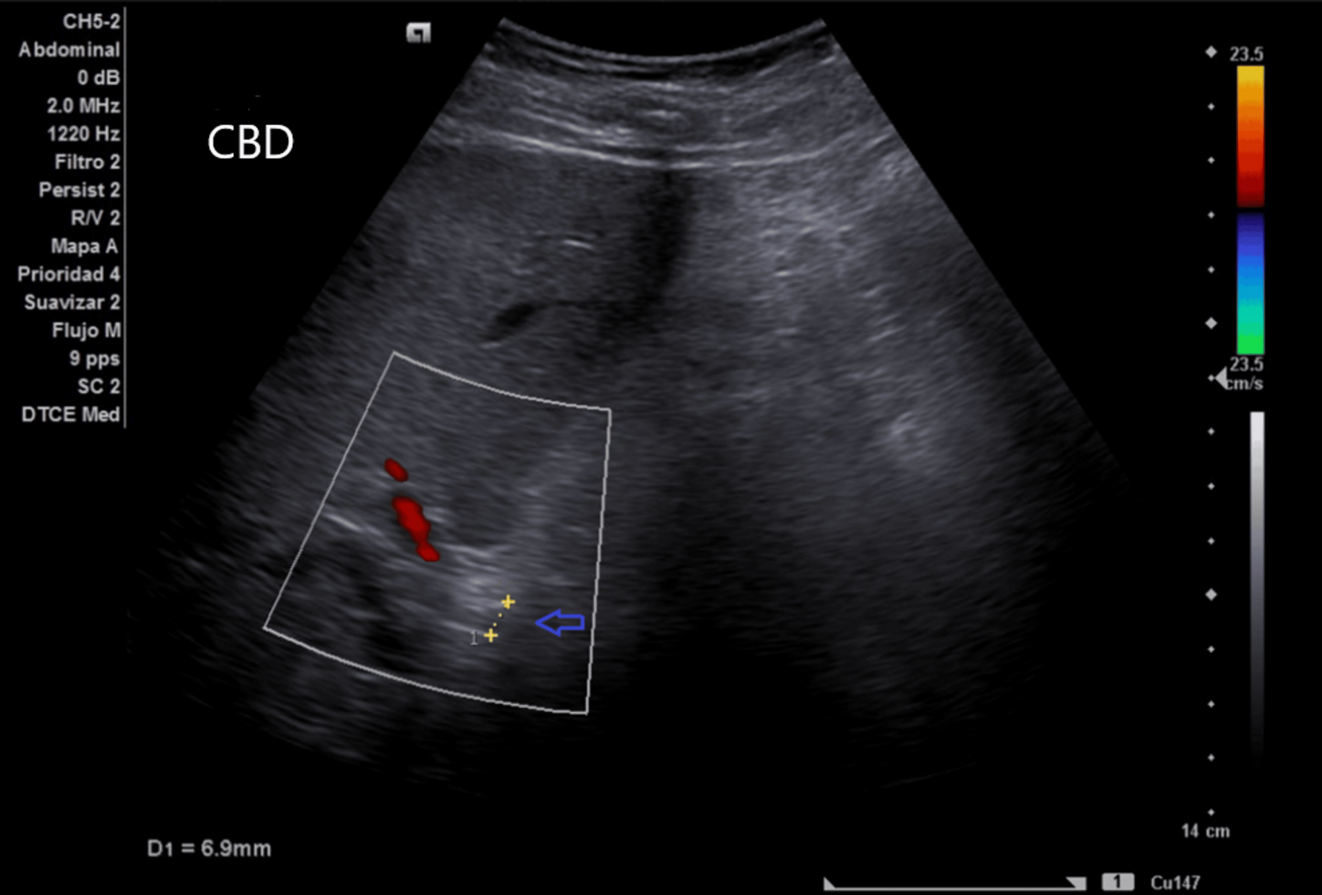
Initial ultrasonography Ultrasound demonstrating CBD dilation up to 1 cm (blue arrow). CBD: common bile duct

**Figure 2:**
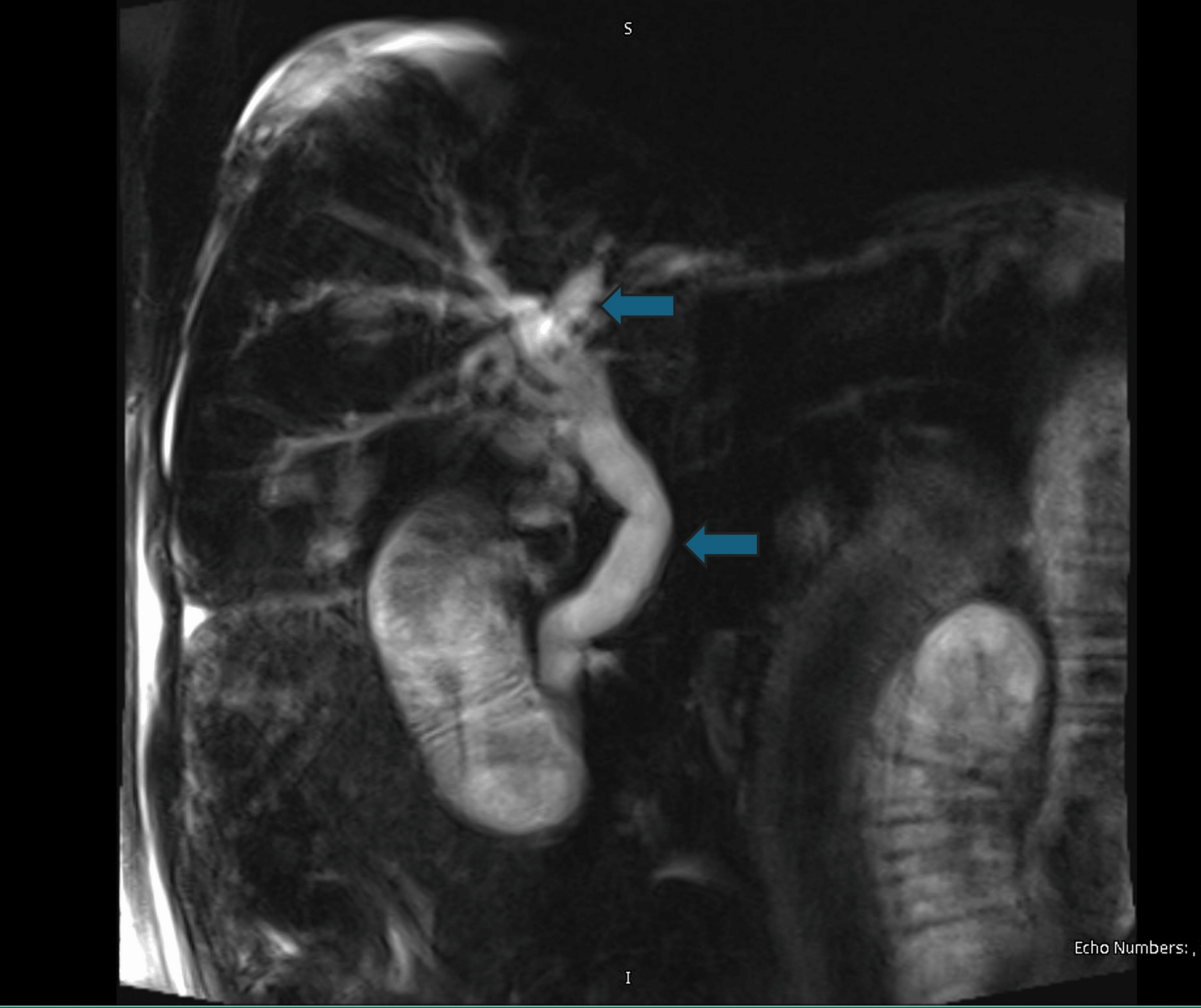
MRCP MRCP revealing the marked dilation of the intrahepatic and extrahepatic biliary tracts (blue arrow). No PDD is identified. MRCP: magnetic resonance cholangiopancreatography; PDD: periampullary duodenal diverticulum

Initial abdominal ultrasonography revealed the postsurgical absence of the gallbladder and dilation of the CBD without intraluminal filling defects (Figure 1). Magnetic resonance cholangiopancreatography (MRCP) confirmed intrahepatic and extrahepatic biliary dilation, with a CBD diameter of approximately 14 mm, absence of choledocholithiasis, and pancreas without alterations (Figure 2). 

**Figure 3:**
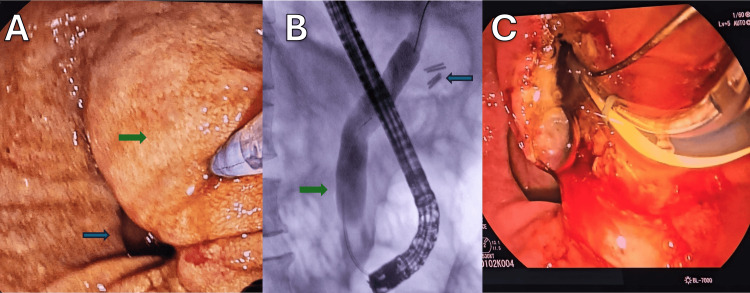
First and second ERCP (A) Inflammatory changes in the ampulla of Vater: endoscopic image during the first ERCP demonstrating the inflammation of the ampullary region (green arrow) with associated PDD (blue arrow). (B) CBD: cannulation of the ampulla revealing a 12 mm dilation of the CBD without stones (green arrow) and evidence of prior cholecystectomy (blue arrow). (C) Sphincterotomy demonstrating the bile flow through the ampulla. ERCP: endoscopic retrograde cholangiopancreatography; PDD: periampullary duodenal diverticulum; CBD: common bile duct

Two sessions of ERCP were performed. During the first procedure, a PDD was identified, with a juxtadiverticular major papilla showing significant inflammation (Figure 3A). A precut sphincterotomy was attempted; however, mild pulsatile bleeding was observed originating from the papillary margin, likely from a small arterial branch within the inflamed periampullary mucosa. The bleeding was limited in volume and was adequately controlled with local epinephrine injection. The intervention was suspended to ensure procedural safety and allow for clinical stabilization before reattempting cannulation.

**Figure 4:**
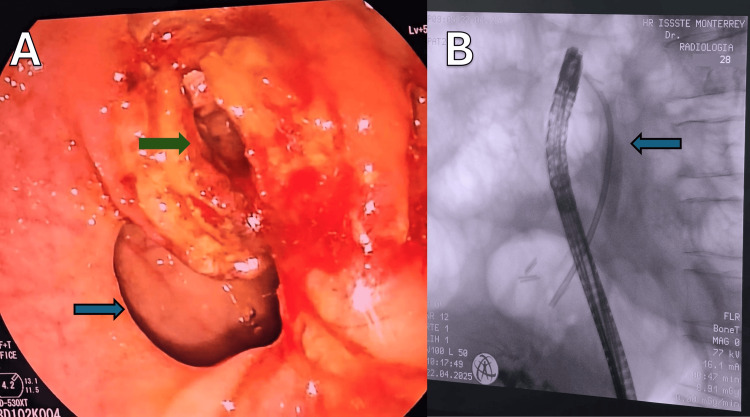
Endoscopic treatment (A) PDD (blue arrow) located less than 2 cm from the ampulla of Vater and complete sphincterotomy (green arrow). (B) Biliary stent: cholangiography with the placement of a 10 Fr biliary stent (blue arrow). Source images could not be digitally exported because the endoscopic system was operated by an external provider without integrated storage; therefore, photographs were taken at the time of the procedure. PDD: periampullary duodenal diverticulum

A second ERCP was performed one week later. Endoscopic exploration of the duodenal diverticulum allowed the identification and cannulation of the ampullary orifice, revealing a dilated CBD measuring approximately 12 mm in diameter (Figure 3B). A complete precut sphincterotomy was performed, releasing biliary material (Figure 3C), and no calculi were observed during the visualization of the biliary tract (Figure 4A). A biopsy of the periampullary mucosa was obtained, and a 10 Fr biliary stent was placed to ensure drainage (Figure 4B). 

The patient was monitored for five days following the procedure and demonstrated progressive laboratory and clinical improvement. He was discharged with a favorable recovery and followed up in the outpatient clinic. Histopathological examination of the periampullary biopsy revealed a chronic inflammatory process, and at follow-up, the patient reported complete resolution of symptoms and normalization of liver function parameters.

## Discussion

Lemmel syndrome is mostly caused by a PDD, and it is defined by the absence of choledocholithiasis or pancreatic neoplasia that could otherwise explain biliary obstruction [3]. Up to 75% of duodenal diverticula are located in the second portion of the duodenum, and they are considered periampullary when situated approximately 2-3 cm from the AV [4]. Although up to 95% of duodenal diverticula remain asymptomatic, between 1% and 5% may become symptomatic or complicated, either with non-pancreatobiliary manifestations such as diverticular perforation or bleeding or with pancreatobiliary complications including cholangitis or pancreatitis [6,7], as observed in our patient who presented with Tokyo Grade III cholangitis.

Three primary mechanisms have been described through which a PDD can cause symptoms: (1) direct compression of the diverticulum on the AV, (2) dysfunction of the sphincter of Oddi, and (3) fibrosis of the major papilla or chronic papillitis secondary to duodenal diverticulitis [8,9]. The third mechanism was considered the most plausible in our patient, given the presence of a deformed ampulla due to chronic inflammatory changes, which was confirmed through histopathological examination.

Imaging studies such as ultrasonography and MRCP can complement case evaluation and may occasionally identify the diverticulum. However, they can sometimes yield misleading diagnoses, such as pancreatic cysts or other pseudomasses. In our case, the duodenal diverticulum was not visualized on imaging studies, which further underscores the diagnostic limitations of non-invasive modalities. Consequently, ERCP remains the gold standard for both diagnosis and treatment in symptomatic patients [9].

Therapeutic options include endoscopic sphincterotomy, with or without stent placement, and, in selected cases, the consideration of percutaneous biliary drainage when endoscopic treatment is not available or surgical treatment such as open diverticulectomy or biliodigestive diversion. Management should be individualized based on the clinical condition and anatomical considerations of each patient [10-12]. In the present case, after clinical optimization and correction of coagulopathy, ERCP was performed due to a high probability of obstructive pathology at the level of the AV, as suggested by a cholestatic pattern with an R factor of 0.5, which led to a successful resolution of the clinical condition. Although PDD is not always visualized on MRCP, the combination of endoscopic visualization, histological confirmation of chronic inflammation, and symptom resolution after sphincterotomy strongly support the diagnosis.

## Conclusions

Lemmel syndrome is a rare clinical entity that poses significant diagnostic challenges due to its nonspecific presentation and broad spectrum of potential complications. Treatment options range from minimally invasive endoscopic approaches to major surgical interventions. Based on a review of the current literature, we emphasize the importance of individualized management, with the therapeutic strategy tailored to the patient's specific condition and underlying pathology. In our case, endoscopic treatment proved successful with complete clinical and biochemical resolution and no need for further intervention.

## References

[1] Lemmel G (1934). Die klinische Bedeutung der Duodenaldivertikel. Archiv für Verdauungskrankheiten.

[2] Bernshteyn M, Rao S, Sharma A, Masood U, Manocha D (2020). Lemmel's syndrome: usual presentation of an unusual diagnosis. Cureus.

[3] Bakula B, Romic I, Sever M, Halle ZB (2021). Duodenal diverticulum causing obstructive jaundice - Lemmel's syndrome. Rev Esp Enferm Dig.

[4] Maloku H, Nuh Aybay M (2023). Periampullary diverticulitis (Lemmel's syndrome) misdiagnosed as pancreatic head tumor: a report of two cases. Int J Surg Case Rep.

[5] Kiriyama S, Kozaka K, Takada T (2018). Tokyo Guidelines 2018: diagnostic criteria and severity grading of acute cholangitis (with videos). J Hepatobiliary Pancreat Sci.

[6] Houssni JE, Cherraqi A, Chehrastane R (2023). Lemmel syndrome: an unusual cause of biliary obstruction secondary to a duodenal juxta-ampullary diverticulum: a report of two cases. Radiol Case Rep.

[7] Colin H, Ndjekembo Shango D, Pilet B, Waignein F, Yengue P (2023). Diverticulitis associated pancreatitis: a report of 2 cases and review of the literature. Acta Gastroenterol Belg.

[8] Farina R, Foti PV, Ilardi A, Basile A (2024). Lemmel's syndrome: lesson based on a case report. J Med Ultrasound.

[9] Battah A, Farouji I, DaCosta TR, Luke ND, Shamoon D, DaCosta T, Bains Y (2023). Lemmel's syndrome: a rare complication of periampullary diverticula. Cureus.

[10] Aslan S, Önder RO (2023). A rare cause of obstructive jaundice and pancreatitis; Lemmel's syndrome. Curr Med Imaging.

[11] Soares CA, Carvalho SA, Duarte ML, Gastaldi TN (2024). Duodenal diverticulum causing Lemmel syndrome: diagnosis by computed tomography. Sultan Qaboos Univ Med J.

[12] Ali IM, Shetty SK, Shetty V (2024). Lemmel syndrome: a surgical enigma. Cureus.

